# Assessment of Seroconversion to SARS-CoV-2 in a Cohort of Pediatric Kidney Transplant Recipients

**DOI:** 10.3389/fped.2020.601327

**Published:** 2020-10-30

**Authors:** Corina Nailescu, Myda Khalid, Amy C. Wilson, Fatima Amanat, Samuel Arregui, Jorge Canas, Jenaya Hooks, Florian Krammer, Andrew L. Schwaderer, David S. Hains

**Affiliations:** ^1^Department of Pediatrics, Indiana University, Indianapolis, IN, United States; ^2^Department of Microbiology, Icahn School of Medicine at Mount Sinai Medical Center, New York, NY, United States

**Keywords:** SARS-CoV-2, COVID-19, pandemic, children, pediatric, kidney, transplant, seroconversion

## Abstract

**Background:** The occurrence of the severe acute respiratory syndrome coronavirus 2 (SARS-CoV-2) and the associated coronavirus disease 2019 (COVID-19) have profoundly affected adult kidney disease patients. In contrast, pediatric solid organ transplant recipients, including pediatric kidney transplant (KT) recipients, do not seem to be at particularly higher risk for SARS-CoV-2 infection or for severe COVID-19 disease. This patient population might be protected by certain mechanisms, such as the immunosuppressive medications with their anti-inflammatory properties or simply being well-versed in self-protection techniques. Assessing SARS-CoV-2 antibody serologies could potentially help understand why this patient population is apparently spared from severe SARS-CoV-2 clinical courses.

**Objective:** To examine SARS-CoV-2 serologic status in a cohort of pediatric KT recipients.

**Methods:** SARS-CoV-2 anti-spike IgG and IgM antibodies were measured by three different methods in pediatric KT recipients coming for routine clinic visits immediately post-confinement in May-June of 2020. The patients were considered seroconverted if SARS-CoV-2 antibodies were positive by 2/3 methods and weak positive/indeterminate if positive by 1/3.

**Results:** Thirty-one patients were evaluated (about 1/3 of our institution's pediatric KT population). One patient seroconverted, while three were considered weak positive/indeterminate. None were symptomatic and none had nasopharyngeal PCR confirmed SARS-CoV-2 disease.

**Conclusions:** Seroconversion to SARS-CoV-2 was rare in this population and likely reflects the social distancing practiced by these patients. The results will serve as a foundation for a future longitudinal study to evaluate the long-term emergence and persistence of antibodies in this population and may inform studies of response to a future vaccine.

## Introduction

The occurrence of the severe acute respiratory syndrome coronavirus 2 (SARS-CoV-2) and the associated coronavirus disease 2019 (COVID-19) have profoundly affected the world's population. Children are less affected and have a milder disease course compared to adults (1). In adults, solid organ transplant (SOT) recipients, including kidney transplant (KT), have been shown to experience more severe outcomes (2, 3). In contrast, pediatric SOT recipients, including KT recipients, do not seem to be at particularly higher risk for SARS-CoV-2 infection or for severe COVID-19 disease (4). This patient population might in fact be protected by certain pediatric specific mechanisms, such as the qualitatively distinct immune responses that children in general have (5), the presence of other simultaneous viruses in the airway mucosa which could compete with SARS-CoV-2 (6, 7), the age-dependent angiotensin-converting enzyme 2 receptor gene expression in nasal epithelium (8) and the lower prevalence of high-risk comorbidities (9). In addition, immunosuppressive drugs with their anti-inflammatory properties could possibly be protective (10), or it may simply be that this population is well-versed in self-protection techniques as a way of life (11). Currently no reports of the SARS-CoV-2 antibody serologies in pediatric KT recipients exist. Addressing this gap in knowledge is important for several reasons: to help recognize why the pediatric KT population is apparently spared from severe COVID-19, to help make further recommendations in regards to school participation in this patient population, to evaluate the long-term emergence and persistence of antibodies in this population, and to inform further studies of response to a vaccine that would hopefully be available in the near future. The goal of this study was to examine SARS-CoV-2 serologic status in a cohort of pediatric KT recipients. Our hypothesis was that pediatric KT recipients would have a low rate of seroconversion to SARS-CoV-2.

## Methods

### Study Population

**Inclusion Criteria:** Patients of any age seen in person in the routine pediatric post-KT clinics at our institution immediately following the end of our state's Stay-At-Home order, in May and June of 2020.

**Exclusion Criteria:** having received blood products in the past 6 months.

### Study Design

The Indiana University Institutional Review Board approved the study. All eligible patients who were scheduled to present in person to the pediatric post-KT clinic for routine laboratory testing and visits during May and June of 2020, immediately after the confinement, were approached over the phone by a study coordinator. Study coordinators discussed the study and consent was obtained from the parent/guardian and assent from the participant. Medical records were used to abstract age, gender, race, time since transplant, type of donor (living vs. deceased), the cause of end-stage renal disease (ESRD), Body Mass Index (BMI) and the presence of rejection episodes in the previous 3 months. During the clinic visit, the patients and their families were carefully interviewed about the presence of possibly SARS-CoV-2 related symptoms, such as sore throat, cough, dyspnea, fever, malaise, myalgias, diarrhea, anosmia, dysgeusia or the presence of the multisystem inflammatory syndrome in children (MIS-C) in the previous 3 months.

### Outcome Measure

Serum IgM and IgG levels were measured utilizing three different SARS-CoV-2 enzyme-linked immunosorbent assays (ELISAs): (1) Abnova (#KA5826) which targets the spike S protein; (2) Indiana University School of Medicine (IUSM) in collaboration with Eli Lilly and Co., Indianapolis, IN, which targets the spike S protein (LSN3832334); (3) Mount Sinai Medical Center's (MSMC) 2-Step ELISA which targets the receptor binding domain (RBD) and spike S protein.

#### Abnova ELISA

Antibody response against the SARS-CoV-2 Spike Protein was measured in human serum using a COVID-19 Human IgG/IgM Assay Kit (Catalog #KA5826 Abnova, Taiwan). Precoated COVID-19 spike protein 96-well plates were washed a single time with 200 μL of Phosphate-buffered saline with 0.1% Tween 20 (PBST) on a shaker gently for 30 s. Following the initial washes, all plates were blocked by adding 200 μL of Ultra Pure 5% Omniblok™ non-fat milk (Catalog #AB10109 American Bio, US) in PBST (w/v) and covered at room temperature (20–24°C) for 1 h. After blocking, the plates were washed again before adding 100 μL of subject serum diluted 1:250 for IgG and 1:2,000 for IgM plates in 2% non-fat milk/PBST. After addition of diluted serum, the plates were covered and allowed to incubate at room temperature for 1 h. After washing five times as previously described, 80 μL of Anti-Human IgG (Catalog #2041-05 Southern Bio, US) and IgM (Abnova, Taiwan), Fc region specific HRP labeled secondary antibody diluted 1:15,000 for IgG and 1:30,000 for IgM in 2% non-fat milk/PBST, were added to the plates. Plates now coated with secondary antibodies were sealed and gently shaken at room temperature for 30 min. The plates were washed 5 times with PBST. Kit provided TMB was prepared and 100 μL of was added to each well and allowed to incubate in the dark for 6 min at 18–25°C. After 6 min, TMB reaction was stopped using 100 μL kit provided Stop Solution and colorimetric reactions were semi-quantitatively measured at Optical Density (OD) 450 nm wavelength in a microplate reader.

#### IUSM in Collaboration With Eli Lilly Anti-spike ELISA

Plates coated overnight with 100 ng/well of SARS-CoV-2 N-terminal domain, SARS-CoV-2 RBD, or SARS-CoV-2 spike AviHis protein (all manufactured by Eli Lilly and Co., Indianapolis, IN). Plates were washed three times with 200 μL of phosphate-buffered saline with 0.1% Tween 20 (PBST). Following the initial washes, all plates were blocked by adding 200 μL of Ultra Pure 2% Omniblok™ non-fat milk (Catalog #AB10109 American Bio, US) in PBST (w/v) and covered at room temperature (20–24°C) for 1 h. After blocking, 100 μL of subject serum diluted 1:500 for IgG were added to the plates in 1% non-fat milk/PBST. After addition of diluted serum, the plates were covered and allowed to incubate at room temperature for 2 h. Upon completion incubation, plates were washed three times with PBST. After washing, 100 μL of Anti-human IgG (Catalog #2041-05 Southern Bio, US, Fc region specific) horseradish peroxidase (HRP) labeled secondary antibody diluted 1:6,000 in 1% non-fat milk/PBST, was added to the plate. Plates incubating with secondary antibody were sealed and incubated for 1 h. The plates were washed three times with PBST. 3,3′,5,5′ tetramethylbenzidine (TMB) was added to each well and allowed to incubate in the dark for 15 min at 18–25°C. TMB reaction was stopped using 100 μL 2 N Phosphoric acid and colorimetric reactions were semi-quantitatively measured at optical density (OD) 450 nm wavelength in a microplate reader.

##### Positive calling

For the Abnova and IUSM developed anti-spike assays, we considered a value greater than the mean of negative control plus 3 times the standard deviation, consistent with standard methodology, intra-plate variability, and with serum values of PCR-confirmed positive control patients (12).

#### MSMC ELISA

For the MSMC assay, previously reported methodology and cut-offs were used (13).

##### Quality measures

Given the differential targets of ELISAs and variable reported sensitivity and specificity by protocol, we determined the threshold for a positive ELISA result for each test based on manufacturers guideline and a large pool of more than 60 historic samples from 2011 (negative controls) and positive controls confirmed on the Roche Cobas commercial platform (14). The sensitivity and specificity the Roche Cobas platform is 92.5 and 100%, respectively. The reported sensitivity and specificity for Abnova ELISA is 70.6 and 95.4%, respectively. For the IUSM in collaboration with Eli Lilly Spike Assay, sensitivity and specificity are 95.5 and 92.3%, respectively.

##### Combined platforms

Due to variation of test characteristics in serologic studies, we considered subjects to have seroconverted if IgG or IgM positive on at least 2 of 3 spike ELISA platforms. If a subject was IgG or IgM positive based on only one method, we considered the result weak positive/indeterminate for seroconversion. When the three tests were combined to optimize testing performance, sensitivity was 94.4% and specificity was 98.5% (15, 16).

## Results

Thirty-one patients were evaluated, which represents ~1/3 of our institution's pediatric KT population, as only patients that presented to our main hospital for in-person follow-up during the study period could be enrolled due to institution-wide COVID-19-related research restrictions in place at the timeAll demographics, characteristics and results are presented in the Table 1. Briefly, the study population had a median age of 12 (range 2–21) years; ~2/3 were male and 3/4 were white. A majority (65%) had congenital anomalies of the kidneys and the urinary tract (CAKUT) as the primary cause of ESRD and were of normal weight (77%). All participants were recipients of their first KT. One patient (3.2%) seroconverted according to our definition, while three (9.7%) were considered weak positive/indeterminate. The one clearly seroconverted patient is Hispanic, but has no other known risk factors for severe COVID-19 disease, such as obesity. None of the patients treated for rejection in the previous 3 months were found to have evidence of seroconversion. It is noteworthy that no patients in this study reported evident symptoms of SARS-CoV-2 infection in the preceding 3 months or at the time of study enrollment. Therefore, none of the seroconverted patients had nasopharyngeal PCR SARS-CoV-2 testing performed.

**Table 1:** Patient characteristics & SARS CoV-2 seroconversion status.

**Patient characteristics**	***n* = 31**
***Age [years; median (range)]***	12 (2–21)
***Sex [male; number (%)]***	21 (68)
***Race [number (%)]***	
Black	3 (10)
Hispanic	5 (16)
White	23 (74)
***Time since transplant [months; median (range)]***	59 (2–150)
***Living donor transplant [number (%)]***	16 (52)
***Cause of ESRD [number (%)]***	
Congenital anomalies of kidney and urinary tract (CAKUT)	20 (65)
Genetic	5 (16)
Acquired inflammatory disease	3 (10)
Other acquired renal disease	3 (10)
***Body mass index (BMI) categorization [number (%)]***	
Obese (BMI ≥ 95th percentile for age and sex)	4 (13)
Overweight (BMI ≥ 85th and <95th percentile for age and sex)	3 (10)
Normal weight (BMI <85th and > 5th percentile for age and sex)	24 (77)
***Treated for rejection in the previous 3 months [number (%)]***	2 (6)
***Symptoms of COVID-19 [number (%)]***	0 (0)
***SARS-CoV-2 seroconversion status [number (%)]***	
Positive	1 (3)
Weak positive/Indeterminate	3 (10)
Negative	27 (87)

## Discussion

Our study found a low prevalence of seroconversion to SARS-CoV-2 in our pediatric KT population during the 2 months immediately following the Indiana Stay-At-Home order during the current pandemic. Additionally, we highlight that the only clearly seroconverted individual was asymptomatic.

There is currently a multitude of tests to assess SARS-CoV-2 antibody levels (13, 17). However, one potential caveat might be cross-reactivity with the seasonal coronaviruses, which could result in false positive testing (18). In addition, the SARS-CoV-2 induced antibody responses are quite variable and possibly not persistent (19, 20). Either false positive or false negative ELISA IgG or IgM results can have important public health ramifications. To improve both sensitivity and specificity of testing, we utilized three different ELISA platforms to measure SARS-CoV-2 anti-spike IgG and IgM antibodies. We only considered participants seroconverted if positive on at least 2 of 3 platforms. This achieved a sensitivity of 94.4% and specificity of 98.5%.

To our knowledge, little is known about the seroconversion status in pediatric patients with SOT, including KT, as the relative impairment of the immune system caused by the anti-rejection medications may affect testing reliability. Data available to date is mixed: a case series of adult SOT recipients showed a positive serologic response in all seven symptomatic hospitalized patients (21), but there is at least one case report of an adult hospitalized KT recipient with clinical and PCR-confirmed SARS-CoV-2 who failed to mount a serologic response, despite being tested for longer than 2 months after the initial presentation (22). In pediatric SOT patients, including KT, even less information exists regarding their serologic status, and this study represents the first report to our knowledge in this population. To place this study in the context of Indiana state level SARS-CoV-2 infection rates at the time of study, one can compare our study's population point prevalence to the statewide one. The Indiana State Department of Health in collaboration with the Indiana University Richard M. Fairbanks School of Public Health conducted a statewide prevalence study utilizing random sampling of Indiana citizens to measure the spread of COVID-19 throughout the state (23). Unfortunately, the Fairbanks study only included subjects age ≥ 12, while in our study the median age was 12, with a range of 2–21 years, so may not be directly comparable. Nevertheless, in the above-mentioned study, the estimated point prevalence as of May 2–3, 2020 in the age group <20 years was 4.3%. This is higher than the point prevalence in our small sample of pediatric KT recipients, which was 1/31 (3.2%).

Evidence thus far has not shown that pediatric SOT recipients suffer worse from COVID-19 compared to other children. However, based on adult data and data regarding other viral infections, the pediatric transplant and infectious disease communities have understandably remained cautious when making recommendations for pediatric SOT recipients in regards to SARS-CoV-2. For example, return to school recommendations for pediatric SOT recipients in the United States during the COVID-19 pandemic have been issued as an expert opinion in order to help families make decisions (24). In these recommendations, pediatric SOT recipients are stratified into high, moderate and low potential risk based on their proximity to their transplant, the presence of recent rejection episodes, clinical stability of their graft function and comorbidities. In addition, local community and school factors must be considered. Our cohort of patients will be studied longitudinally. Eventually, seroconversion could be yet another factor in favor of in person school participation for a particular patient, in cases when it is difficult for the family to make decisions. However, at this point, until more evidence is available, one cannot and should not make a firm statement regarding either going back to school in person or the need for self-isolation, solely based on having serological evidence of prior SARS-CoV-2 infection.

Identifying asymptomatic patients who might have developed serological responses to SARS-CoV-2, such as in our study, could be important in investigating responses to a future vaccine. Once such patients are identified, SARS-CoV-2-specific CD4+ T cell memory could be assessed specifically prior to vaccination, followed by measurement of neutralizing antibodies following vaccination. Such experiments would offer an invaluable opportunity to ascertain the potential biological significance of pre-existing SARS-CoV-2-reactive T cells (25).

We acknowledge that our study has limitations. As with many pediatric SOT studies, our study is limited by its relatively small sample size and the setting of a single pediatric KT program, where only about 1/3 of the population was captured In addition, the cross-sectional design eliminates any ability to comment on long-term antibody persistence.

In conclusion, seroconversion to SARS-CoV-2 was rare in this cohort of pediatric KT recipients and likely reflects the social distancing practiced by these patients. Continuing to study this cohort will be important to evaluate the long-term emergence and persistence of antibodies as our patients return to school and other activities. In addition, it may inform studies of response to a future vaccine.

## Data Availability Statement

The original contributions presented in the study are included in the article/supplementary materials, further inquiries can be directed to the corresponding author/s.

## Ethics Statement

The studies involving human participants were reviewed and approved by Indiana University Institutional Review Board. Written informed consent to participate in this study was provided by the participants' legal guardian/next of kin.

## Author Contributions

CN was responsible for study design, patient recruitment, data gathering and analysis, and manuscript writing. MK helped with study design and actively participated in patient recruitment, and manuscript revisions. AW helped with study design and actively participated in manuscript writing. FA actively participated in generating laboratory data, data analysis and interpretation. SA, JC, and JH participated in generating laboratory data, data collection, as well as reviewing the manuscript. FK and AS participated in concept and design, review of the manuscript, as well as obtaining funding. DH had full access to all the data in the study and takes responsibility for the integrity of the data, participated in manuscript review, and mentored CN. All authors contributed to the article and approved the submitted version.

## Conflict of Interest

AS reported receiving grants from Eli Lilly Foundation and the National Institutes of Health. FK reported that a patent to The Icahn School of Medicine at Mount Sinai Medical Center is in the process of licensing assays based on the assays described herein to commercial entities and that the school of medicine has filed for patent protection, pending, and licensed. The remaining authors declare that the research was conducted in the absence of any commercial or financial relationships that could be construed as a potential conflict of interest.
